# Effect of Different Exercise Loads on Testicular Oxidative Stress and Reproductive Function in Obese Male Mice

**DOI:** 10.1155/2020/3071658

**Published:** 2020-01-27

**Authors:** Xuejie Yi, Donghui Tang, Shicheng Cao, Tao Li, Haining Gao, Tie Ma, Tingting Yao, Jing Li, Bo Chang

**Affiliations:** ^1^Exercise and Health Research Center/Department of Kinesiology, Shenyang Sport University, Shenyang, 110102 Liaoning, China; ^2^PE College of Beijing Normal University, Beijing 100875, China; ^3^Department of Sports Medicine, School of Public and Basic Sciences, China Medical University, Shenyang, 110122 Liaoning, China

## Abstract

This study is aimed at investigating the effect of different exercise loads on the reproductive function of obese male mice and the underlying mechanisms. Male mice with high-fat diet-induced obesity were divided into obesity control (OC), obesity moderate-load exercise (OME), and obesity high-load exercise (OHE) groups. The OME and OHE groups were subjected to swimming exercise 5 days per week over a duration of 8 weeks, with the exercise load progressively increased to 2 h per day in the OME group and 2 h twice per day in the OHE group. In the OC group mice without exercise regimen, we observed a decrease in mRNA expression of antioxidant enzymes, increase in free radical products, upregulation of mRNA and protein expression of nuclear factor-*κ*B and proinflammatory cytokines, inhibition of mRNA and protein expression of testosterone synthases, decrease in the serum testosterone level and sperm quality, and increase in sperm apoptosis. Although both moderate-load exercise and high-load exercise reduced body fat, only moderate-load exercise effectively alleviated obesity-induced oxidative stress, downregulated the expression of nuclear factor-*κ*B and proinflammatory cytokines, and reversed the decrease in mRNA and protein expression of testosterone synthases, serum testosterone level, and sperm quality. These changes were not observed in the OHE group mice. Obesity-induced testicular oxidative stress and inflammatory response decreased testosterone synthesis and sperm quality. Moderate-load exercise alleviated the negative effect of obesity on male reproductive function by decreasing testicular oxidative stress and inflammatory responses. Although high-load exercise effectively reduced body fat, its effects on alleviating oxidative stress and improving male reproductive function were limited.

## 1. Introduction

Over the last four decades, the number of people with obesity worldwide has increased rapidly from 105 million in 1975 to 641 million in 2014 [1]. In addition, infertility rates have increased parallelly with obesity rates [2, 3]. In some countries with high obesity incidence, monitoring of the total sperm count and sperm motility in males indicated an annual decrease of 1.5% [4, 5]. Increasing evidence suggests that obesity damages reproductive health in males and causes late-onset male hypogonadism [6, 7], which is characterized by low serum testosterone levels and relevant symptoms (poor libido, erectile dysfunction, diminished sperm quality parameters, and reproductive dysfunction) [8–10]. The mechanisms through which obesity affects male reproductive function are complex. Previous reports indicate that oxidative stress [11] and inflammatory responses [12–14] are associated with impaired function of Leydig cells. Furthermore, according to human [15] and animal studies [16], when oxidative stress and inflammatory responses in the semen of obese males are increased, the sperm motility is reduced, morphological defects are increased, and DNA damage and apoptotic rate of germ cells are increased [17]. However, it remains unclear whether there is a correlation between obesity, oxidative stress, and inflammatory response.

The effects of exercise on weight loss and body fat reduction are well-known, and exercise load is known to be positively correlated with body fat reduction; however, reports about the effects of exercise-mediated body fat reduction on improvement in male reproductive function are inconsistent [18, 19]. We have previously reported that 8 weeks of moderate- or high-load exercise effectively reduced body fat, but the negative effects of obesity on male reproductive function were alleviated only by moderate-load exercise and not by high-load exercise [20]. Studies have shown that exercise load is closely related to oxidative stress [21]. Low-load exercise does not cause oxidative stress injury. Moderate-load exercise increases free radicals associated with an increased oxygen intake; as a positive adaptive response, it can also stimulate the expression and activity of antioxidant enzymes and enhance the body's antioxidant capacity [22]. However, due to an increased oxygen consumption during the heavy-load exercise, a large number of free radicals is produced through various mechanisms. Since the free radicals accumulate in excess, it exceeds the body's ability to resist oxidative stress, attacks biological macromolecules and membrane structures, and causes oxidative damage to the body that could be linked to exercise-related hypoandrogenemia and diminished sperm quality [23]. Therefore, we hypothesized that the inconsistencies in the effect of different exercise loads are related to oxidative stress and the inflammatory response. This study provides an experimental basis to further determine the mechanisms by which exercise and obesity affect male reproductive function, and it also provides a theoretical basis to develop effective prevention methods.

## 2. Materials and Methods

### 2.1. Animals

Fifty male C57BL/6L mice (age, 4 weeks; weight, 16-19 g) were purchased from Beijing Vital River Laboratory Animal Technology Co., Ltd. (Beijing, China) under permit number SCXK (Beijing) 2016-0006. All mice were housed under controlled experimental conditions (22 ± 5°C, 50 ± 10% relative humidity, and 12 h light/12 h dark cycle) and provided with food and water ad libitum. Each cage contained no more than five mice. All animal experiments in this study were carried out in accordance with the National Institutes of Health Guide for the Care and Use of Laboratory Animals and approved by the Animal Ethics Committee of Shenyang Sport University.

### 2.2. Obese Mouse Model

Ten mice were randomly selected to form a normal control (NC) group that was provided a normal diet (ND), while the remaining 40 mice were fed with a high-fat diet (HFD). The ND and HFD were formulated according to previously reported nutrient formulations [16], and the feed formulations were provided by Jianmin Company Ltd. (Shenyang, China). After 10 weeks of feeding, six obesity-resistant mice were eliminated from the HFD group, while the remaining mice reached body weights of more than 120% of the mean body weight of the NC group mice, thereby satisfying the criteria for an obese animal model [16]. The mice that satisfied the criteria were stratified according to their body weight and randomly assigned to the following three groups: obesity control (OC), 10 mice; obesity moderate-load exercise (OME), 12 mice; and obesity high-load exercise (OHE), 12 mice. Differences in the body weights of mice within the three groups were not significant (*P* > 0.05).

### 2.3. Exercise Intervention

The mice in the OME and OHE groups were subjected to 8 weeks of exercise intervention, which involved free swimming without interference in a plastic pool of diameter of 45 cm, water depth 60 cm, and water temperature of 32 ± 1°C. A previously described exercise program [16] was adopted, which consisted of 2 days of acclimatization training followed by 8 weeks of proper swimming training. The exercise load was progressively increased during the training period, with an initial duration of 20 min once per day in the OME group and 20 min twice per day (6 h interval between the two sessions) in the OHE group. During weeks 1 and 2, the training time was increased in increments of 10 min until reaching 120 min per day and 120 min twice per day at the end of week 2 in the OME and OHE groups, respectively. These exercise loads were maintained for the subsequent 6 weeks of training.

### 2.4. Sample Collection

To observe the adaptive responses of mice to long-term exercise, sample collection was performed 36–40 h after the last exercise session in the OME group and the OHE group. The mice in both groups were fasted for 12 h before sample collection to eliminate the effect of exercise-induced stress responses and diet on the various indicators. Each mouse was weighed and subsequently anesthetized by intraperitoneal injection of pentobarbital (50 mg/kg body weight; Sinopharm Chemical Reagent Co., Ltd., Shanghai, China). Blood samples were collected from the orbital venous plexus and centrifuged for 20 min (4°C, 900 *g*) to separate the serum, which was stored at -80°C before serum testosterone testing. Simultaneously, rapid separation of the testis and epididymis was performed, and the sperm count, motility, and apoptosis rate in the epididymis were measured and assessed [16]. The separated testis was immersed in liquid nitrogen for rapid freezing and stored at -80°C until further use. The peritesticular, perirenal, and mesenteric fat was separated and weighed on an electronic balance to determine the abdominal fat content of each mouse.

### 2.5. Cauda Epididymal Sperm Count and Motility Measurements

The epididymis was removed from one side of the mice and placed in 1.0 mL of HEPES buffer. The epididymis was then cut at the junction between the corpus and the cauda epididymis, and the cauda was placed in a well containing 1.0 mL of HEPES buffer. The epididymis was cut into several segments with a pair of scissors and then gently pressed to release the semen from the vas deferens to mixing with the buffer. The number of sperm per microliter was recorded using a hemocytometer (15 *μ*L per side). Sperm count and motility were assessed in accordance with the World Health Organization guidelines (≥200 sperms were counted per sample). The sperm count was determined using a hemocytometer. Sperm motility was assessed blindly under a light microscope by classifying 200 sperms per animal as either progressive motile, nonprogressive motile, or immotile. Motility was expressed as percentage of total motile sperm (progressive motile and nonprogressive motile sperms) [16].

### 2.6. Hormone Measurement

Samples for concentration measurement of serum total testosterone were processed using a commercial enzyme-linked immunosorbent assay (ELISA) kit according to the manufacturer's protocol, and their absorbance was measured using a Multiskan GO 1510 (Thermo Fisher Scientific, Waltham, MA, USA). The ELISA kit was supplied by R&D Systems (Minneapolis, MN, USA). The detection limit of the testosterone kit was 0.75–24 ng/mL. The intra-assay coefficient of variation (CV) was less than 10%, and the interassay CV was less than 15% for the ELISA kit. All measurements were conducted in the Key Laboratory of Exercise Science of Shenyang Sport University.

### 2.7. Measurement of Sperm Apoptosis

After membrane removal, the other epididymis of each mouse was cut into pieces and incubated in saline at 37°C for 10 min to disassociate the sperm. The sample was centrifuged at 400 *g* for 5 min after filtering, and then the supernatant was discarded to collect the cells. Phosphate-buffered saline was added to form a sperm suspension, and 5 *μ*L of Annexin V-fluorescein isothiocyanate and 5 *μ*L of propidium iodide were added. The suspension was then mixed gently and incubated at 20°C away from light for 10 min. Measurements were performed using a flow cytometer (CytoFLEX; Beckman Coulter, Brea, CA, USA) within 1 h, with a minimum of 10000 spermatozoa examined during each measurement. Forward scatter/side scatter gating was adopted to eliminate disturbances from cell debris and cell aggregation. After sorting the spermatozoa and cell debris using scatter signals, the live, dead, and cells were distinguished on a bivariate fluorescent signal scatter plot. The excitation wavelength used was 488 nm; green fluorescence (480–530 nm) was detected using the FL1 channel, while red fluorescence (580–630 nm) was detected using the FL2 channel. The positive cell rate and mean fluorescence intensity were analyzed using CellQuest software (BD Biosciences, Franklin Lakes, NJ, USA). The percentage of early apoptotic sperm relative to the total sperm count was calculated for each group.

### 2.8. Determination of Oxidative Stress in Testicular Tissue

Testicular homogenate of 5% or 10% was prepared and centrifuged. Subsequently, the following indicators with reference to the product specifications of Nanjing Jiancheng Bioengineering Institute were determined using the supernatant: nitric oxide (NO; nitrate reductase method), nitric oxide synthase (NOS; colorimetric method), reduced glutathione (GSH), glutathione peroxidase (GSH-PX; colorimetric method), malondialdehyde (MDA; TBA method), catalase (CAT; visible light), hydrogen peroxide (H_2_O_2_), total superoxide dismutase (T-SOD; hydroxylamine method), and total antioxidant capacity (ABTS method).

### 2.9. Isolation of RNA and Real-Time PCR Analysis

An RNA extraction agent (Vazyme Biotech Co., Ltd., Nanjing, China) was used to extract total RNA from the testis of each mouse according to the manufacturer's instructions. Subsequently, a reverse transcription kit (Promega, Madison, WI, USA) was used for the reverse transcription of 1 *μ*g of total RNA to cDNA in a 96-well thermal cycler (Applied Biosystems, Foster City, CA, USA). The target mRNA content was measured in a real-time PCR amplification system (Applied Biosystems) using a real-time PCR amplification kit (Promega) in accordance with the manufacturer's instructions. All primers were designed and synthesized by Sangon Biotech (Shanghai) Co., Ltd. (Shanghai, China). The primer sequences for the target genes are shown in Table 1. Each sample was amplified in triplicate; GAPDH was used as the housekeeping gene, and the 2^-*ΔΔ*Ct^ method was used to calculate the relative expression levels.

### 2.10. Western Blotting

After weighing the separated testis from each mouse, RIPA lysis buffer and phenylmethylsulfonyl fluoride, a protease inhibitor, were added, and the testis tissue was cut into pieces and homogenized in an ice bath. The homogenate was centrifuged, and the supernatant was removed for protein quantification in a microplate reader (Thermo Fisher Scientific) using the BCA protein assay kit (Beijing Dingguo Changsheng Biotechnology Co., Ltd., Beijing, China). The target protein was separated by sodium dodecyl sulfate gel electrophoresis; 30–50 *μ*L of protein lysis buffer was added to each well. The separated target protein and the internal control *β*-actin were transferred onto a nitrocellulose membrane and blocked for 1 h using 5% nonfat dry milk blocking buffer. After adding the primary antibody (rabbit anti-mouse; ABclonal, Wuhan, China), the nitrocellulose membrane containing the target protein was incubated overnight (12 h) at 4°C. The target proteins included NF-*κ*B (ABclonal, A2547), tumor necrosis factor- (TNF-) *α* (11948; Cell Signaling Technology, Danvers, MA, USA), IL-1*β* (12426; Cell Signaling Technology), IL-10 (5261; Cell Signaling Technology), SF-1 (10976; Santa Cruz Biotechnology, Dallas, TX, USA), StAR (58013; Abcam, Cambridge, UK), P450scc (175408; Abcam), and *β*-actin (sc-1496; Santa Cruz Biotechnology). Subsequently, the membrane was incubated at 20°C for 1 h with the fluorescent dye-labeled secondary antibody (IRDye@ 800CW-labeled goat anti-rabbit; LI-COR, Lincoln, NE, USA) at 1 : 15000 dilution. Finally, the nitrocellulose membrane was inserted into the Odyssey infrared imaging system (LI-COR) to quantitatively analyze the protein bands using Image Studio imaging software provided with the system. The final result was reported as the target protein content/*β*-actin content ratio [16].

### 2.11. Statistical Analysis

The data are expressed as mean ± SE. Multiple group comparisons were performed by one-way analysis of variance (one-way ANOVA) followed by a Student-Newman-Keuls post hoc test to conduct multiple comparisons. The results were considered significant for *P* values of <0.05. These analyses were performed using SPSS 18.0 software (SPSS Inc., Chicago, IL, USA).

## 3. Results

### 3.1. Effect of HFD and Exercise on Body Weight and Abdominal Fat Content

**Figure 1:**
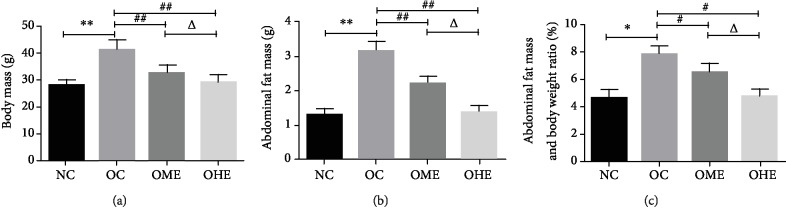
Effect of high-fat diet and exercise on body weight and abdominal fat content. Data are mean ± SE; NC: normal control; OC: obesity control; OME: obesity moderate exercise; OHE: obesity high exercise, vs. NC: ^∗^*P* < 0.05, ^∗∗^*P* < 0.01; vs. OC: ^#^*P* < 0.05, ^##^*P* < 0.01; vs. OME: ^△^*P* < 0.05, ^△△^*P* < 0.01.

After 18 weeks of high-fat diet feeding, the body weight (Figure 1(a)), abdominal fat content (Figure 1(b)), and liposome ratio of the OC group (Figures 1(b) and 1(c)) were significantly higher than those of the NC group. After 8 weeks of exercise intervention, the body weight (Figure 1(a)), abdominal fat content (Figure 1(b)), and lipid ratio of the OME and OHE groups were significantly lower than those of the OC group; the decrease in the OHE group was higher than that in the OME group (Figures 1(a)–1(c)).

### 3.2. Effects of Obesity and Exercise on Testosterone Level and Sperm Quality

**Figure 2:**
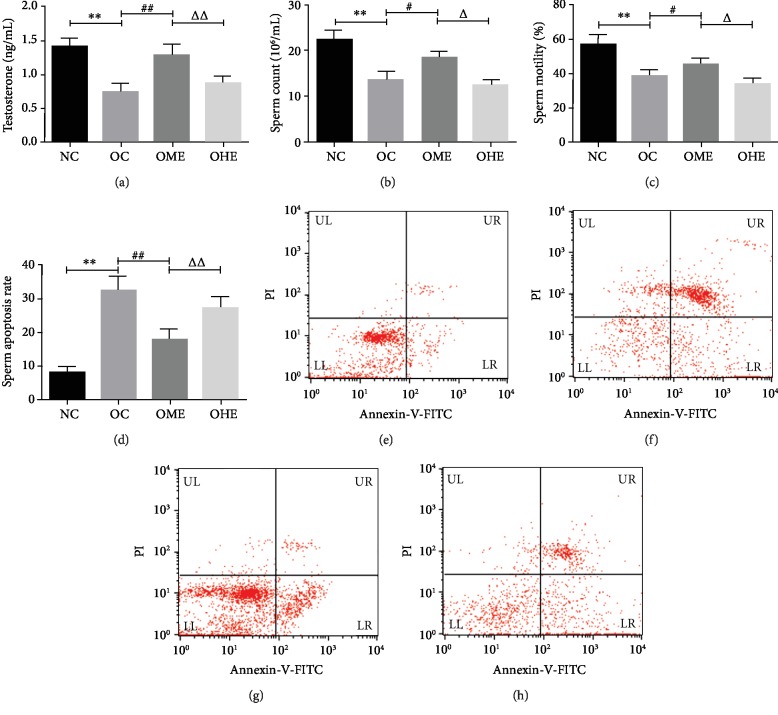
Effects of obesity and exercise on testosterone level and sperm quality. Data are mean ± SE; NC: normal control; OC: obesity control; OME: obesity moderate exercise; OHE: obesity high exercise. (e) Normal control (NC) group; (f) high-fat diet obesity control (OC) group; (g) obesity moderate-load exercise (OME) group; (h) obesity high-load exercise (OHE) group. Upper left (UL) region: necrotic cells; upper right (UR) region: late apoptotic cells; lower left (LL) region: live cells; lower right (LR) region: early apoptotic cells. vs. NC: ^∗^*P* < 0.05, ^∗∗^*P* < 0.01; vs. OC: ^#^*P* < 0.05, ^##^*P* < 0.01; vs. OME: ^△^*P* < 0.05, ^△△^*P* < 0.01.

Compared with those in the NC group, the OC group had a significantly decreased serum testosterone level (Figure 2(a)), sperm count (Figure 2(b)), and sperm activity (Figure 2(c)), along with a significantly increased sperm apoptosis rate (Figures 2(d) and 2(f)). After 8 weeks of exercise intervention, the serum testosterone level (Figure 2(a)), sperm count (Figure 2(b)), and sperm motility of mice (Figure 2(c)) in the OME group were significantly increased, while the sperm apoptosis rate was decreased (Figures 2(d) and 2(g)). The serum testosterone level (Figure 2(a)), sperm count (Figure 2(b)), sperm motility (Figure 2(c)), and sperm apoptosis rate (Figures 2(d) and 2(h)) of the OHE group did not change significantly but were significantly higher than those of the OME group (Figures 2(a)–2(d) and 2(h)).

### 3.3. Effect of High-Fat Diet and Exercise on Testicular Oxidative Stress

**Figure 3:**
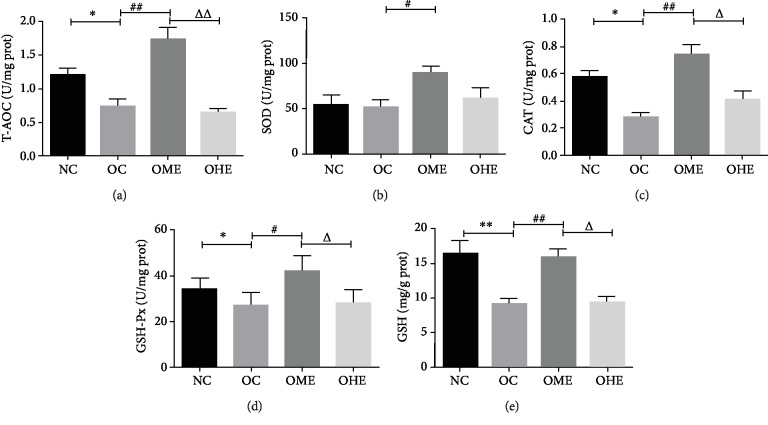
Influence of high-fat diet and exercise on the testicular antioxidant system. T-OAC: total antioxidant capacity; SOD: superoxide dismutase; CAT: catalase; GSH-Px: glutathione peroxidase; GSH: glutathione. Data are mean ± SE; NC: normal control; OC: obesity control; OME: obesity moderate exercise; OHE: obesity high exercise, vs. NC: ^∗^*P* < 0.05, ^∗∗^*P* < 0.01; vs. OC: ^#^*P* < 0.05, ^##^*P* < 0.01; vs. OME: ^△^*P* < 0.05, ^△△^*P* < 0.01.

**Figure 4:**
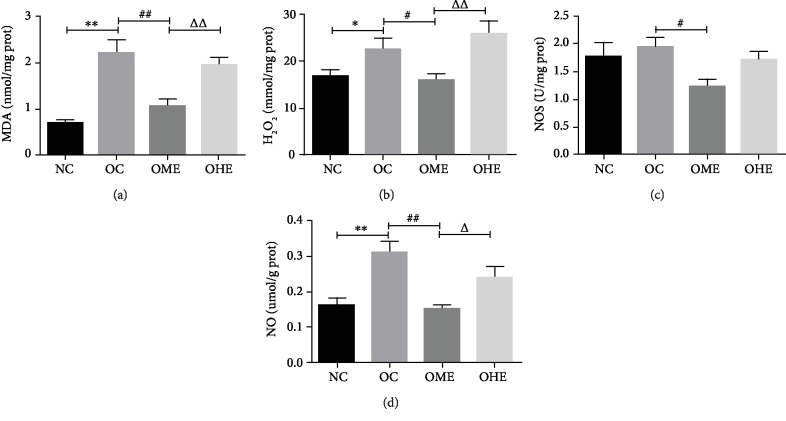
Effect of high-fat diet and exercise on the testicular oxidative stress product. MDA: malondialdehyde; H_2_O_2_: hydrogen peroxide; NOS: nitric oxide synthase; NO: nitric oxide. Data are mean ± SE; NC: normal control; OC: obesity control; OME: obesity moderate exercise; OHE: obesity high exercise, vs. NC: ^∗^*P* < 0.05, ^∗∗^*P* < 0.01; vs. OC: ^#^*P* < 0.05, ^##^*P* < 0.01; vs. OME: ^△^*P* < 0.05, ^△△^*P* < 0.01.

**Figure 5:**
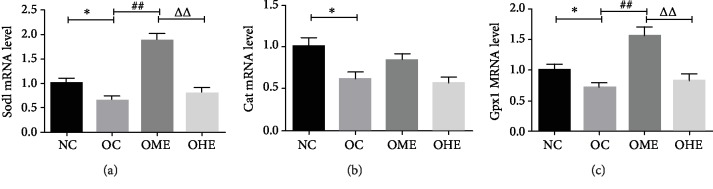
Effect of obesity and exercise on the mRNA expression of antioxidant enzymes. T-OAC: total antioxidant capacity; SOD: superoxide dismutase; CAT: catalase; GSH-Px: glutathione peroxidase. Data are mean ± SE; NC: normal control; OC: obesity control; OME: obesity moderate exercise; OHE: obesity high exercise, vs. NC: ^∗^*P* < 0.05, ^∗∗^*P* < 0.01; vs. OC: ^#^*P* < 0.05, ^##^*P* < 0.01; vs. OME: ^△^*P* < 0.05, ^△△^*P* < 0.01.

Compared with those of the NC group, the antioxidant indexes T-AOC, CAT, GSH-Px, and GSH of the testis tissue of the OC group decreased significantly (Figures 3(a) and 3(c)–3(e)) and the oxidative stress products MDA, H_2_O_2_, and NO increased significantly (Figures 4(a)–4(d)). After 8 weeks of exercise intervention, the antioxidant indexes T-AOC, SOD, CAT, GSH-Px, and GSH of the OME group showed a significant recovery (Figures 3(a)–3(e)) and oxidative stress products MDA, H_2_O_2_, NOS, and NO showed a significant regression (Figures 4(a)–4(d)), but these changes did not occur in the OHE group (Figures 3(a)–3(e) and 4(a)–4(d)), and the values of most those indicators were significantly different from those of the OME group (Figures 3(a)–3(e) and 5(a)–5(d)).

### 3.4. Effect of Obesity and Exercise on the mRNA Expression of Antioxidant Enzymes

The mRNA expression of SOD and GSH-Px in the testicular tissues of the OC group was significantly lower than of the NC group (Figures 5(a)–5(c)). After 8 weeks of moderate-load exercise, the mRNA expression of SOD and GSH-Px in the OME group showed a significant recovery (Figures 5(a)–5(c)). In the OHE group, there were no significant changes in the three antioxidant enzymes (Figures 5(a)–5(c)); there was a significant difference in the mRNA expression of SOD and GSH-Px between the OHE and OME groups (Figures 5(a)–5(c)).

### 3.5. Effect of Obesity and Exercise on mRNA and Protein Expression of NF-*κ*B, TNF-*α*, IL-1, and IL-10 in the Testicular Tissue

**Figure 6:**
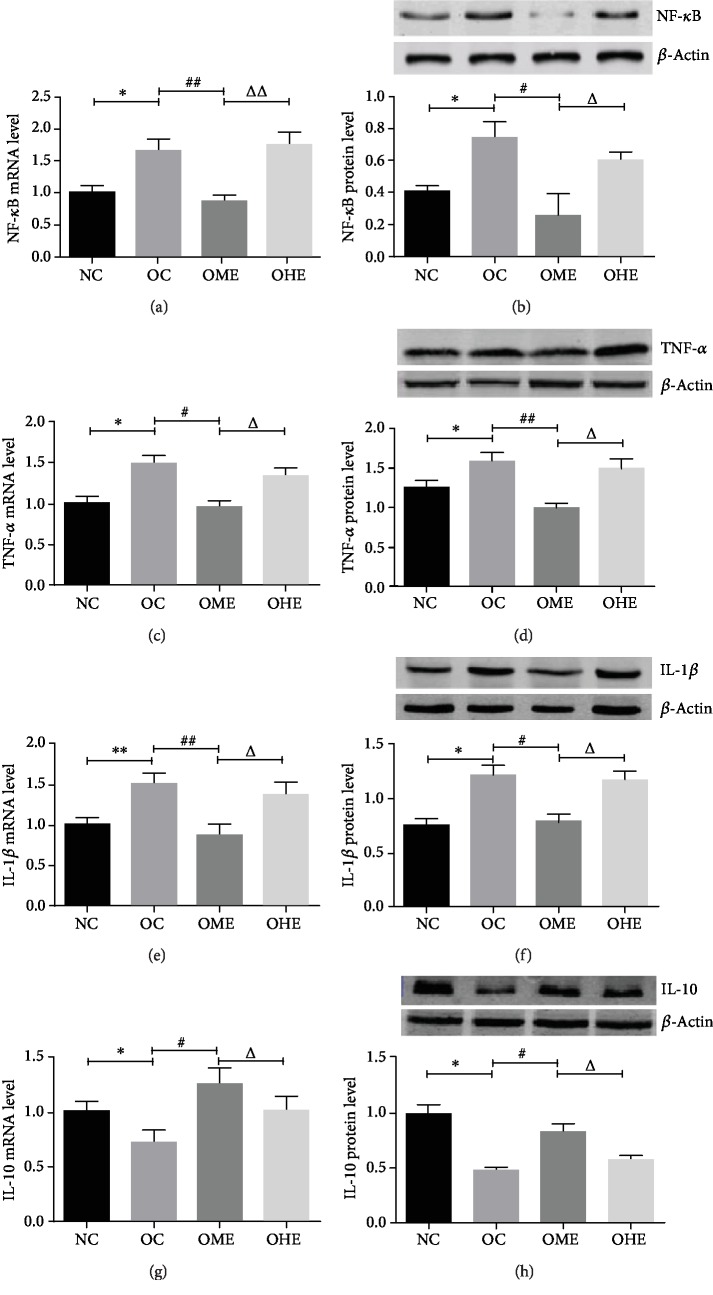
Effect of obesity and exercise on the mRNA level and protein expression of NF-*κ*B, TNF-*α*, IL-1*β*, and IL-10 in the testicular tissue. NF-*κ*B: nuclear factor *κ*B; TNF-*α*: tumor necrosis factor-*α*; IL-1*β*: interleukin-1*β*; IL-10: interleukin-10. Data are mean ± SE; NC: normal control; OC: obesity control; OME: obesity moderate exercise; OHE: obesity high exercise; vs. NC: ^∗^*P* < 0.05, ^∗∗^*P* < 0.01; vs. OC: ^#^*P* < 0.05, ^##^*P* < 0.01; vs. OME: ^△^*P* < 0.05, ^△△^*P* < 0.01.

In the testicular tissue of the OC group, the mRNA and protein expression of NF-*κ*B, TNF-*α*, and IL-1 increased significantly (Figures 6(a)–6(f)), while the mRNA and protein expression of the anti-inflammatory cytokine IL-10 decreased significantly (Figures 6(g) and 6(h)). Compared with that of the OC group, the mRNA and protein expression of NF-*κ*B, TNF-*α*, and IL-1 in the OME group decreased significantly (Figures 6(a)–6(f)), while the mRNA and protein expression of IL-10 increased significantly (Figures 6(g)–6(h)). In the OHE group, there were no significant changes in the mRNA and protein expression of NF-*κ*B, TNF-*α*, IL-1, and IL-10 (Figures 6(a)–6(h)); however, there were significant differences between the expression levels in the OME and OHE groups (Figures 6(a)–6(h)).

### 3.6. Effect of Obesity and Exercise on mRNA and Protein Levels of SF-1, StAR, and P450scc in the Testicular Tissue

**Figure 7:**
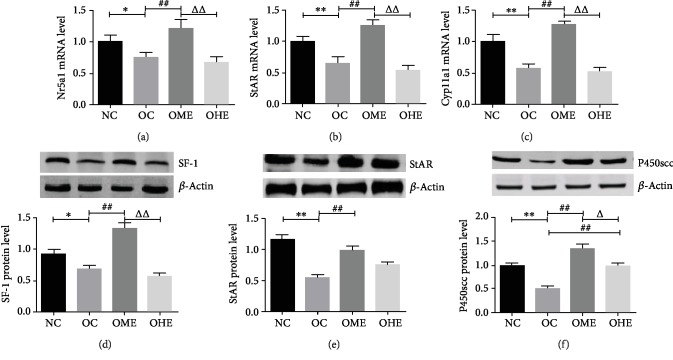
Influence of obesity and exercise on mRNA and protein expression of SF-1, StAR, and P450 in the testicular tissue. SF-1: steroidogenic factor-1; StAR: steroidogenic acute regulatory protein; and P450scc: P450 side chain cleavage (Cyp11a1). Data are mean ± SE; NC: normal control; OC: obesity control; OME: obesity moderate exercise; OHE: obesity high exercise, vs. NC: ^∗^*P* < 0.05, ^∗∗^*P* < 0.01; vs. OC: ^#^*P* < 0.05, ^##^*P* < 0.01; vs. OME: ^△^*P* < 0.05, ^△△^*P* < 0.01.

Figure 7 shows that the mRNA and protein levels of SF-1, StAR, and P450 in the OC group were significantly lower than those in the NC group (Figures 7(a)–7(f)). The mRNA and protein levels of SF-1, StAR, and P450scc increased significantly in the OME group (Figures 7(a)–7(f)), while those in the OHE group were not significantly changed (Figures 7(a)–7(f)). There were significant differences in the mRNA expression of SF-1, StAR, and P450 and protein levels of SF-1 and P450 between the OME and OHE groups (Figures 7(a)–7(f)).

## 4. Discussion

To understand the mechanisms by which exercise affects reproductive function in men with obesity, we conducted a serial of experiments. Our previous studies indicated that both long-term moderate- or high-load exercise could effectively reduce body fat and alleviate leptin resistance. Interestingly, only the moderate-load exercise could alleviate the negative effects of obesity on the male reproductive function [20]. We hypothesized that this phenomenon might be related to the oxidative stress and the inflammatory response, as supported by our results, which are as follows: male obesity disrupted the balance between oxidation and antioxidation in the testicular tissue, induced oxidative stress, upregulated NF-*κ*B, and triggered the inflammatory response, which reduced testosterone biosynthesis and sperm quality, thereby negatively affecting male reproductive function. Moderate-load exercise effectively alleviated the high oxidative stress induced by obesity, downregulated the expression of NF-*κ*B and proinflammatory cytokines, and improved testosterone biosynthesis and sperm quality. However, high-load exercise did not alleviate the levels of obesity-induced oxidative stress and inflammatory response in the testicular tissue and did not significantly improve the reduced male reproductive function. Therefore, the oxidative stress-inflammatory response triggered by high-load exercise may have offset the inhibitory effects of body fat reduction on oxidative stress. Therefore, it is speculated that variations in exercise regimens have different effects on the male reproductive function caused by obesity via the inhibition/stimulation of oxidative-stress inflammatory response.

In addition to being one of the main factors affecting male infertility [24, 25], oxidative stress is closely related to obesity and exercise [26, 27]. Studies have shown that oxidative stress markers are positively correlated with the body mass index and body fat percentage [28]. Increased oxidative stress induced by excessive fat accumulation is an early promoter and key pathogenic mechanism of obesity-related metabolic syndrome [29]. Obesity can trigger systemic oxidative stress [30], which includes testicular and sperm oxidative stress, thereby resulting in the reduction of testosterone synthesis, spermatogenesis, and sperm quality [15, 26]. This study had similar results, that is, in obese male mice, the serum testosterone levels were reduced; sperm quality parameters were decreased; sperm apoptosis was increased; NO, NOS, H_2_O_2_, and MDA levels in the testicular tissue were significantly increased; T-AOC concentration was decreased; catalase and GSH activities and mRNA expression were decreased; and SOD mRNA expression was significantly decreased, although the SOD activity did not change significantly. The mechanisms through which obesity induces oxidative stress in testicular tissue remain unclear. A previous study showed that elevated levels of glucose and free fatty acids led to an increase in mitochondrial ROS. Obesity induces excessive accumulation of lipids in adipocytes, which causes an increase in the substrate load in the mitochondria, promotes the expression of NADPH oxidase subunits, and leads to increased ROS, reduced SOD and GSH-PX activities, and increased oxidative stress in the mitochondria [31]. In obese mice, immunohistochemical results revealed increases in the number of Leydig cells, the number and volume of lipid droplets in the cells [10], and the level of MDA, an oxidative stress marker and lipid peroxidation product. Among the membrane structures of Leydig cells, the mitochondria and endoplasmic reticulum are rich in polyunsaturated fatty acids, which are highly prone to ROS attacks, resulting in the production of large amounts of MDA [32–34]. Obesity causes excessive accumulation of lipids in the body. For instance, in the Leydig cells of obese mice, the number and volume of lipid droplets are increased [10], causing an increase in the substrate load in the mitochondria, promoting the production of ROS in the mitochondria, and reducing the activity of SOD and GSH-PX [31]. Free radicals, when accumulated in excess, attack the unsaturated fatty acids (PUFA) in the membrane of mitochondria and endoplasmic reticulum, producing a large amount of MDA [33, 34]. The toxicity of MDA induces a decrease in the cholesterol synthesis, cholesterol transfer, and steroid synthesis capabilities of the endoplasmic reticulum, ultimately resulting in reduced testosterone synthesis and spermatogenesis [10, 35]. This association is corroborated by our result that the mRNA and protein levels of SF-1, StAR, and P450 in the testis tissue, along with the serum testosterone levels, were significantly lower in the OC group than in the NC group. Similarly, the sperm membrane surface and DNA molecules are rich in unsaturated fatty acids [35] that are also prone to ROS attacks, which generates large amounts of lipid peroxidation products. The lipid peroxidation products can harm the membrane integrity, fluidity, and permeability, as well as damage DNA structures and accelerate cell apoptosis, thereby resulting in increased defective sperm counts and reduced sperm motility [17]. These negative effects influence sperm capacitation and the acrosome reaction, thereby affecting the fertilization ability of the sperms [36]. Our experiment showed that the sperm quality parameters decreased and the apoptosis increased in the OC group. Therefore, obesity-induced fat accumulation in the testicular tissue triggered oxidative stress, inhibited testosterone synthesis and spermatogenesis, and reduced sperm quality, thereby negatively affecting obese male reproductive function.

Obesity-induced oxidative stress causes dysregulated expression of inflammation-related adipokines in the adipose tissue [29], which is promoted by the inflammatory signal transcription factor NF-*κ*B that plays a key role in oxidative stress-induced dysregulation of adipokine expression and is recognized as a major mediator of oxidative stress-induced signal transduction in adipose cells [37, 38]. Studies have shown that ROS can activate I*κ*B kinase, which promotes the degradation of I*κ*B proteins. This results in the release of NF-*κ*B dimers that translocate into the nucleus and control the gene transcription of certain proinflammatory cytokines (IL-1*β*, IL-6, TNF-*α*, and IL-8) [39]. Addition of the antioxidant *N*-acetyl cysteine has shown to impair NF-*κ*B activation and inhibit TNF-*α* [40]. However, other studies have reported that proinflammatory cytokines, such as IL-1*β* and TNF-*α*, can inhibit the gene expression of testosterone synthases StAR, 3*β*-HSD, and P450c17 via the activation of NF-*κ*B, which results in decreased testosterone synthesis within Leydig cells [13, 14]. Based on these findings, it was concluded that cytokines, such as TNF-*α* and IL-1*β*, simultaneously act as the downstream targets and stimulants of NF-*κ*B, thereby activating NF-*κ*B and further causing a continuous and magnified inflammatory response [41, 42]. In this study, we observed that obesity induced an increase in oxidative stress in the testicular tissue, which simultaneously increased the mRNA and protein levels of NF-*κ*B, TNF-*α*, and IL-1; decreased the mRNA and protein levels of the anti-inflammatory cytokine IL-10; and decreased the mRNA and protein levels of key testosterone synthases SF-1, StAR, and P450. Therefore, a long-term high-fat diet induced the production of large amounts of ROS in the testes, activating NF-*κ*B, triggering the inflammatory response, and inhibiting testosterone biosynthesis [16, 17], which may be one of the main mechanisms for decreased serum testosterone levels in obese male mice.

In addition to reducing the mass of white adipose tissue (WAT), exercise training can also reduce oxidative stress in these tissues. Farias et al. [43] found that exercise training induced a reduction in the expression of the NAPDH oxidase NOX2 in the WAT and increased the enzyme activity of Mn-SOD, thereby reducing oxidative damage [43, 44]. However, very few studies have examined the effect of exercise on oxidative stress in the testicular tissue. In this study, the MDA, H_2_O_2_, NOS, and NO levels were significantly reduced in the testicular tissues of obese mice, whereas the T-AOC levels and activities and the mRNA expression of SOD, GSH, and catalase were significantly increased after 8 weeks of moderate-load exercise intervention. These results are consistent with the increased testosterone levels and improved sperm quality achieved by moderate-load exercise. However, these effects were not observed after high-load exercise; this may be related to the exercise load, which is closely associated with oxidative stress. It has been established that the oxygen demand increases during exercise and the oxygen consumption in the skeletal muscles increases by more than 100-fold compared to sedentary conditions; moreover, under exercise conditions, the free radical levels also increase [21]. On the other hand, the increases in free radicals can stimulate increased antioxidant enzyme activities, thereby preventing cell damage caused by excessive free radical production [22]. The effect of this positive adaptive response on the male reproductive function is typically manifested as a significant increase in the serum testosterone level and the quality, count, and DNA integrity of the sperm [45]. However, excessive exercise load leads to the production of large amounts of free radicals that exceed the body's antioxidative capacity; this excess of free radicals can induce damage to the male reproductive function. In addition, studies have shown that the testicular tissues of male rats subjected to strenuous exercise exhibited increased oxidative stress levels, decreased antioxidant enzyme activities, decreased levels of the key steroidogenic enzymes, and decreased testosterone synthesis and spermatogenesis, indicating a correlation between strenuous exercise-induced oxidative stress and reproductive dysfunction [23, 46]. In this study, oxidative stress, testosterone synthase expression, serum testosterone levels, sperm quality, and sperm apoptosis rate in the OHE group were not effectively improved compared with those in the OC group. Thus, oxidative stress induced by high-load exercise may have offset the protective effects of fat reduction against oxidative stress. Nevertheless, the molecular mechanisms underlying this hypothesis are not clear. Next, to find further evidence for this hypothesis, we measured the expression of cytokines related to the inflammatory response.

Interestingly, the expression analysis of testicular tissue in obese mice revealed that moderate-load exercise was associated with decreased mRNA and protein levels of NF-*κ*B, IL-1*β*, and TNF-*α*, along with increased mRNA and protein levels of the anti-inflammatory cytokine IL-10. These observations are consistent with the changes in oxidative stress markers, mRNA and protein expression of the key testosterone synthesis enzymes (SF-1, StAR, and P450), serum testosterone level, and sperm quality parameters. Our findings in the OME group were similar to the results described in a study by Zhao et al. [45], which demonstrated that early-life or lifelong appropriate exercise effectively alleviated age-induced oxidative damage in the testes and downregulated the expression of the proinflammatory cytokines IL-1*β* and TNF-*α* and the inflammatory signaling pathway component NF-*κ*B but increased the levels of the anti-inflammatory cytokine IL-10, enhancing testosterone biosynthesis, serum testosterone levels, and sperm quality parameters. In addition, previous *in vitro* studies had established that TNF-*α* and IL-1*β* could inhibit testosterone synthesis in rats by inhibiting the mRNA and protein expression of P450scc [47], 17 *α*-hydroxylase/17,20-lyase (P450c17), and 3*β*-hydroxysteroid dehydrogenase in rat Leydig cells [48]. Conversely, IL-10 can inhibit the synthesis of the proinflammatory cytokines TNF-*α*, IL-1*α*, and IL-1*β* [45]. Based on these previous findings and the outcomes in this study, it is suggested that long-term moderate-load exercise can inhibit the expression of proinflammatory cytokines by reducing oxidative stress and simultaneously promoting testosterone synthesis by enhancing anti-inflammatory cytokine expression.

In this study, we found that long-term high-load exercise did not significantly improve the mRNA and protein expression of NF-*κ*B and proinflammatory cytokines in the testicular tissues of obese mice, which was consistent with our data for the oxidative stress, the testosterone synthesis factors SF-1, StAR, and P450, and the sperm quality. However, another study showed that high-load exercise training increased the levels of IL-1*β*, IL-6, IL-8, and TNF-*α* in the seminal plasma [49]. An earlier study in adult males also showed that after lipopolysaccharide stimulation, long-term high-load exercise training reduced the numbers of blood monocytes, neutrophils, and dendritic cells, along with decreases in the synthesis of IL-1*β*, IL-6, TNF-*α*, and macrophage inflammatory protein-1*β* [50]. The discrepancies between our results and those of previous studies may be related to the differences in the study subjects. Because of ectopic lipid deposition, the testicular tissues of obese male mice are in a state of high oxidative stress and high inflammatory response [10]. Although high-load exercise can reduce whole-body lipid deposition and ectopic lipid deposition, thereby reducing energy overload in the mitochondria and alleviating excessive ROS production [51], the high-load exercise can also increase the synthesis of free radicals [52], offsetting these positive effects in obese subjects. A limitation of this study was that the relationship between oxidative stress and inflammation in reproduction was not confirmed *in vivo* by injection of an antioxidant. Assessing this relationship will provide a theoretical basis to develop treatments for improving the reproductive function in men with obesity. This aspect will be further explored.

## 5. Conclusions

Long-term high-fat diet induces obesity, causing excessive ectopic deposition of lipids, triggering oxidative stress in the testis tissue, possibly triggering inflammatory response via NF-*κ*B, and reducing testosterone biosynthesis and sperm quality. Moderate-load exercise can be effective in lowering body fat, alleviating obesity-induced, high oxidative stress in the testis tissue, downregulating the expression of NF-*κ*B and proinflammatory cytokines, increasing the testosterone biosynthesis, and improving the sperm quality. Although the high-load exercise is better in reducing body fat, it has a negligible effect on reversing the high oxidative stress, the inflammatory response in the testis tissue, the testosterone biosynthesis, and the sperm quality in obese male mice. Overall, different exercise regiments may have different effects on the male reproductive function caused by obesity through the inhibition/stimulation of oxidative-stress inflammatory response.

## Figures and Tables

**Table 1:** Primer sequences for real-time PCR.

Gene	Forward primer	Reverse primer
*Cat*	5′-ATTGCCGTCCGATTCTCC-3′	5′-CCAGTTACCATCTTCAGTGTAG-3′
*Gpx1*	5′-CAGTTCGGACATCAGGAGAA-3′	5′-AGAGCGGGTGAGCCTTCT-3′
*Sod1*	5′-ACTTCGAGCAGAAGGCAAGC-3′	5′-GTCTCCAACATGCCTCTCTTCAT-3′
*Nf-kb*	5′-ACCTGAGTCTTCTGGACCGCTG-3′	5′-CCAGCCTTCTCCCAAGAGTCGT-3′
*Tnfα*	5′-ACGGCATGGATCTCAAAGAC-3′	5′-GTGGGTGAGGAGCACGTAGT-3′
*IL1β*	5′-GCTGCTTCCAAACCTTTGAC-3′	5′-AGCTTCTCCACAGCCACAAT-3′
*Il10*	5′-CTGTCATCGATTTCTCCCCTGTG-3′	5′-TGGTCTTGGAGCTTATTAAAATCAC-3′
*Nr5a1*	5′-TTGGGTCAGAGGTCATCCTT-3′	5′-CAACAGTGGACTTCCTGCTTC-3′
*Star*	5′-TGCCGAAGACAATCATCAAC-3′	5′-CAGGTCAATGTGGTGGACAG-3′
*Cyp11a1*	5′-CCTTTATGAGATGGCACACAA-3′	5′-GATGCTGGCTTTGAGGAGTG-3′
*Gapdh*	5-GACAACTTTGG-CATTGTGGA-3′	5′-ATGCAGGGATGATGTTCTGG-3′

## Data Availability

The data used to support the findings of this study are available from the corresponding author upon request.
